# Supplementary material for: Tomographic Reconstruction of Quasistatic Surface Polariton Fields
Source: ACS Photonics. 2022 Dec 14;10(1):185–96. doi: 10.1021/acsphotonics.2c01431 (PMC9853846; doi:10.1021/acsphotonics.2c01431)
Supplement: Supplementary file 1 — ph2c01431_si_001.pdf [file ph2c01431_si_001.pdf]

Tomographic reconstruction of quasistatic surface polariton fields
Supporting Information

Raphael Hauer,[†] Georg Haberfehlner,[†] Gerald Kothleitner,[†,‡] Mathieu Kociak,[¶] and Ulrich Hohenester[*,§]

†*Graz Centre for Electron Microscopy, Steyrergasse 17, 8010 Graz, Austria*

‡*Institute for Electron Microscopy and Nanoanalysis, Graz University of Technology, Steyrergasse 17, 8010 Graz, Austria*

¶*Université Paris-Saclay, CNRS, Laboratoire de Physique des Solides, 91405 Orsay. France*

§*Institute of Physics, University of Graz, Universitätsplatz 5, 8010 Graz, Austria*

E-mail: ulrich.hohenester@uni-graz.at

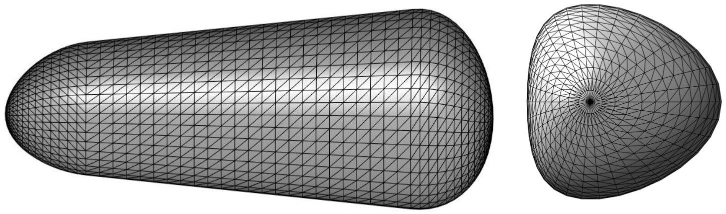

Figure S1: Boundary triangulation of squeezed rod. As an additional example for our tomography scheme, we consider a nanorod with reduced symmetry where the rod is squeezed in all axes directions. (left) side view and (right) top view.

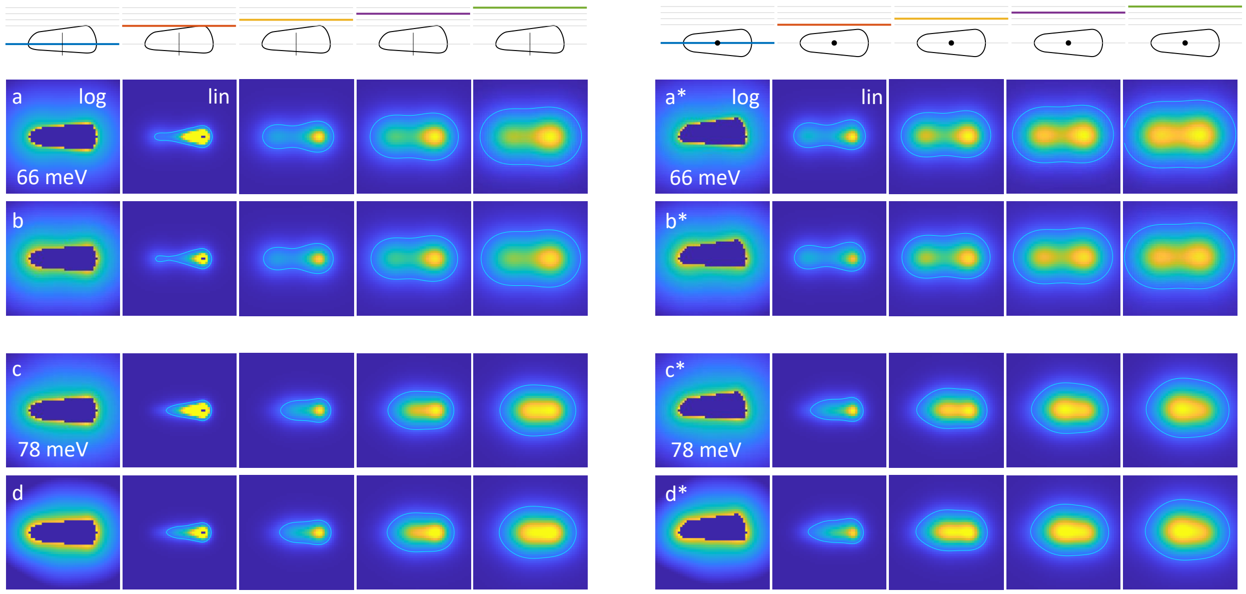

Figure S2: Same as Fig. 7 of main text, however, for the squeezed rod shown in Fig. S1. The (a,b) dipole and (c,d) quadrupole resonances are at 66 meV and 78 meV respectively. The planes where the LDOS is computed are shown on top of the figure. As a basis we use the Laplace-Beltrami modes for the squeezed rod, the optimization is performed with 50 eigenmodes and using the approach with the orthogonal matrix. The agreement between simulated (a,c) and reconstructed (b,d) LDOS maps is very good throughout.

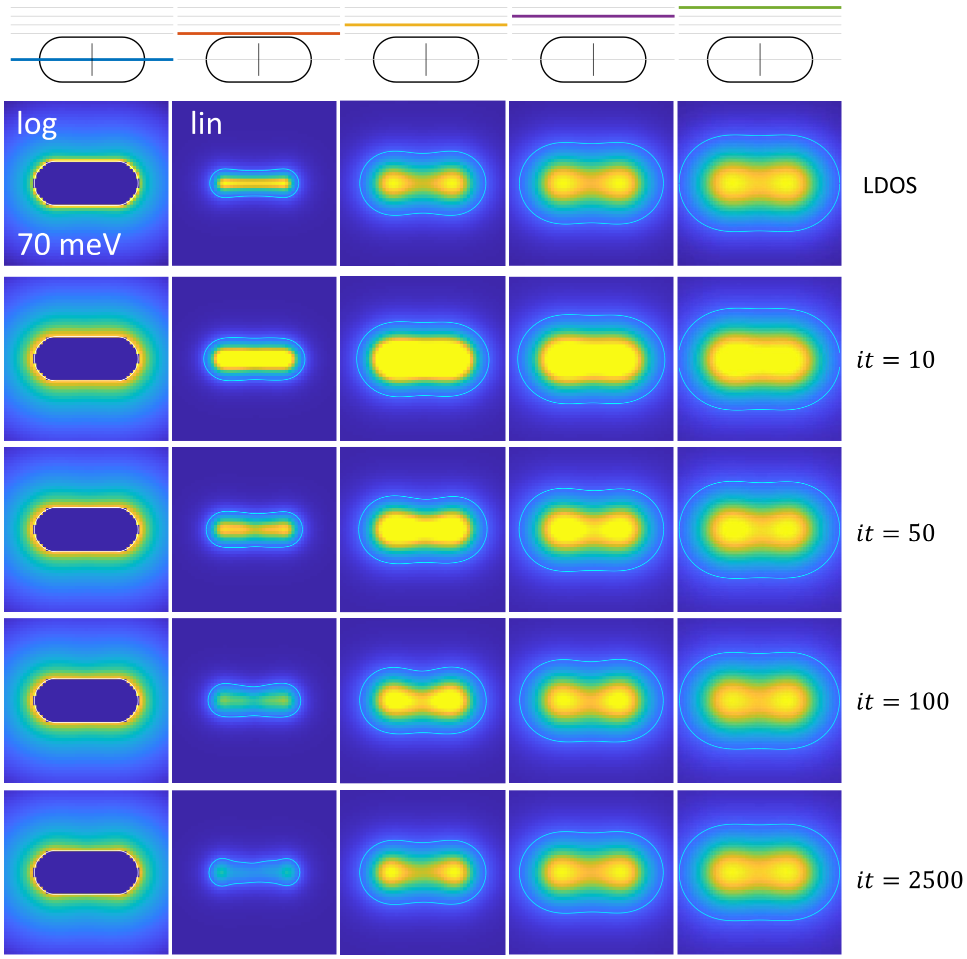

Figure S3: Same as Fig. 5(a,b) of main text for smooth nanorod, however, terminating the optimization after a fixed number of iterations `it` (see right-hand side of panels). In the reconstruction we use 50 modes and the approach with the orthogonal matrix. The main features of the LDOS are reconstructed after a relatively small number of iterations, say a few tens, whereas the amplitudes L_k need a relatively large number of iterations until convergence. The results in the last row correspond to the converged results.

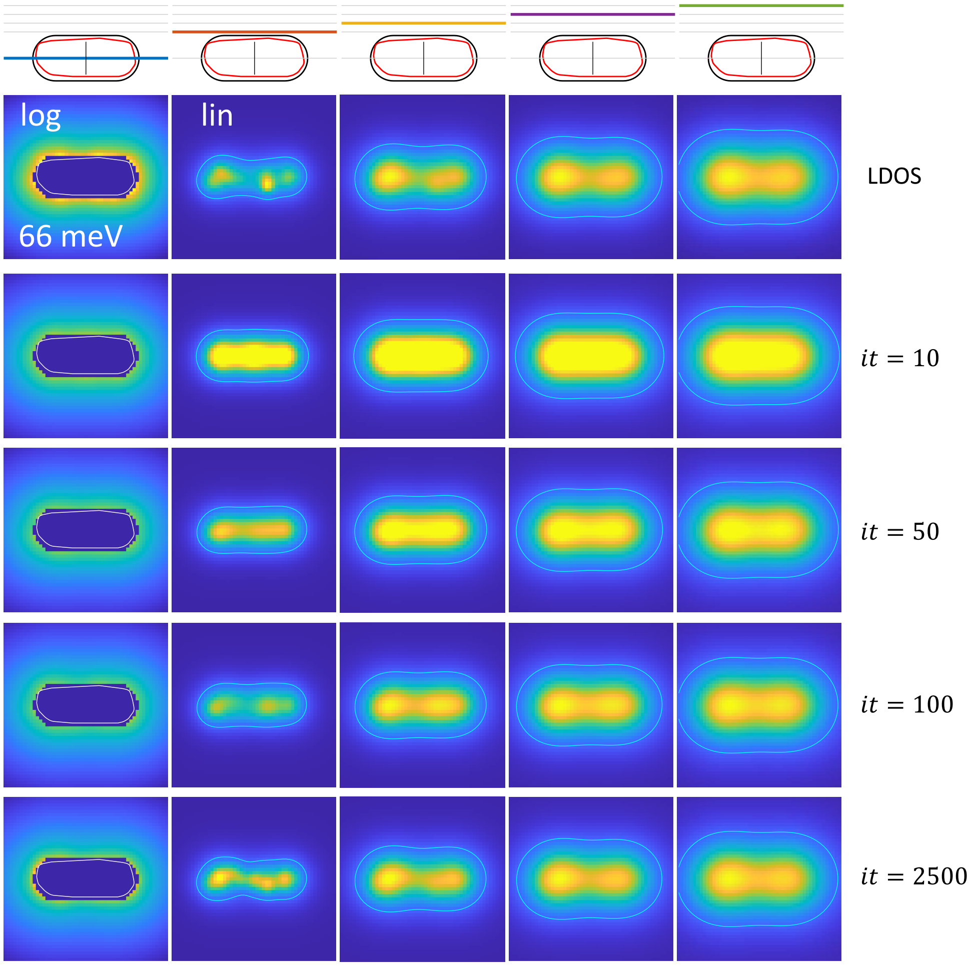

Figure S4: Same as S3, but for rough nanorod. We use $(n, m) = (100, 10)$ and the approach for the full matrix.

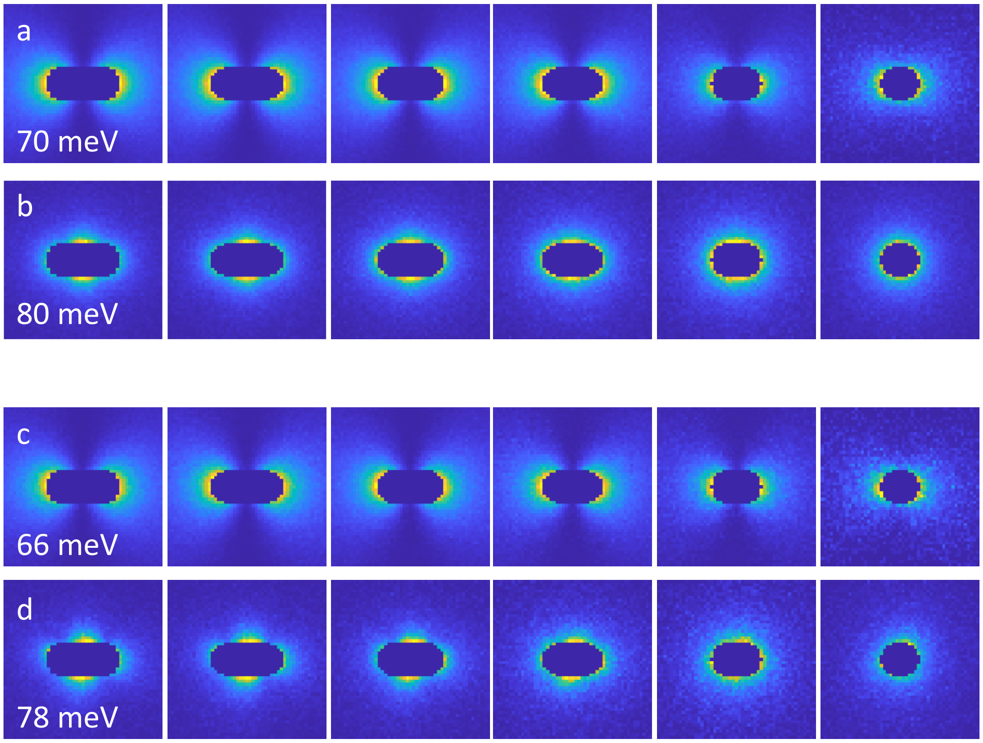

Figure S5: Simulated EELS maps with artificial noise added. We consider noise with a Poissonian distribution that is added using the MATLAB command `imnoise(I,'poisson')` with a signal-to-noise ratio of 10 : 1. Panels (a,b) show the rotated EELS maps for the (a) dipole and (b) quadrupole resonance of the smooth rod. Panels (c,d) show the rotated EELS maps for the (d) dipole and (d) quadrupole resonance of the rough rod.

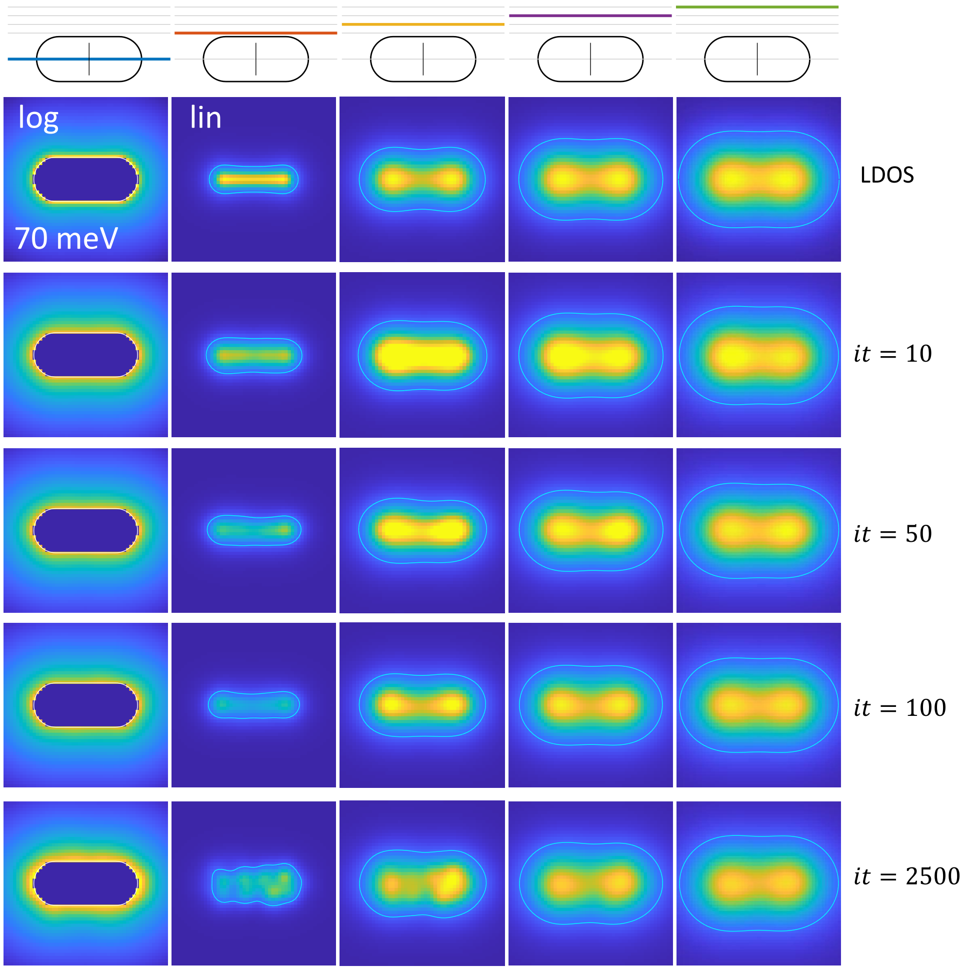

Figure S6: LDOS reconstruction for smooth nanorod and using the noisy EELS maps of Fig. S5(a,b). We investigate the maps as a function of optimization iterations, see also Fig. S3. The reconstructed maps agree well with the simulated ones after say 100 iterations, with increasing iterations the LDOS maps in close vicinity to the rod deteriorate in presence of noise.

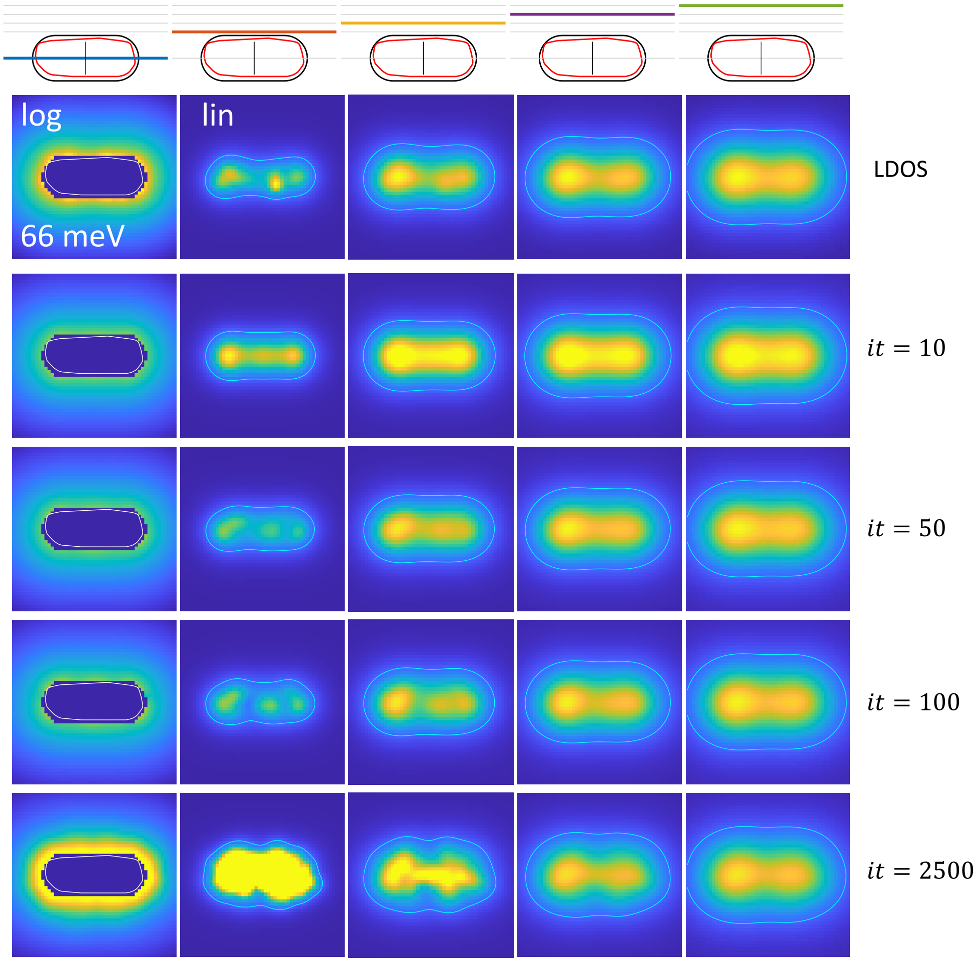

Figure S7: LDOS reconstruction for rough nanorod and using the noisy EELS maps of Fig. S5(c,d). See also captions of Figs. S4 and S6.
